# Integrated genomics and proteomics of the *Torpedo californica *electric organ: concordance with the mammalian neuromuscular junction

**DOI:** 10.1186/2044-5040-1-20

**Published:** 2011-05-04

**Authors:** Suzanne E Mate, Kristy J Brown, Eric P Hoffman

**Affiliations:** 1 Department of Biochemistry and Molecular Genetics, IBS, George Washington University, Washington DC, USA; 2 Department of Pediatrics, George Washington School of Medicine, Washington DC, USA; 3 Research Center for Genetic Medicine, Children's National Medical Center, Washington DC, USA; 4 Department of Integrative Systems Biology, George Washington School of Medicine, Washington DC, USA

## Abstract

**Background:**

During development, the branchial mesoderm of *Torpedo californica *transdifferentiates into an electric organ capable of generating high voltage discharges to stun fish. The organ contains a high density of cholinergic synapses and has served as a biochemical model for the membrane specialization of myofibers, the neuromuscular junction (NMJ). We studied the genome and proteome of the electric organ to gain insight into its composition, to determine if there is concordance with skeletal muscle and the NMJ, and to identify novel synaptic proteins.

**Results:**

Of 435 proteins identified, 300 mapped to *Torpedo *cDNA sequences with ≥2 peptides. We identified 14 uncharacterized proteins in the electric organ that are known to play a role in acetylcholine receptor clustering or signal transduction. In addition, two human open reading frames, C1orf123 and C6orf130, showed high sequence similarity to electric organ proteins. Our profile lists several proteins that are highly expressed in skeletal muscle or are muscle specific. Synaptic proteins such as acetylcholinesterase, acetylcholine receptor subunits, and rapsyn were present in the electric organ proteome but absent in the skeletal muscle proteome.

**Conclusions:**

Our integrated genomic and proteomic analysis supports research describing a muscle-like profile of the organ. We show that it is a repository of NMJ proteins but we present limitations on its use as a comprehensive model of the NMJ. Finally, we identified several proteins that may become candidates for signaling proteins not previously characterized as components of the NMJ.

## Background

Ionic gradients across cell membranes (bioelectricity) are utilized by all organisms. Some fish have developed extreme adaptations of bioelectricity with the evolution of electric organ systems. It is thought that electric organs have evolved independently six or seven times in fish and can be classified as either weak or strong, which is reflective of the size and function of the organs within the fish. For example, *Gymnotids *are weakly electrogenic and only possess accessory electric organs used for electroreception and electrolocation [1]. In contrast, *Torpedinid *and *Electrophorous *are strongly electrogenic and possess organs that account for approximately one-third of the organism's mass and are used for generation of electric shocks for predation or protection [2].

Developmental studies have shown most electric organs are derived from muscle anlage tissue; the exception is the neurogenic development of the *Sternarcus *electric organ. Several basic differences exist amongst myogenic-derived electric organs. The location of the myogenic-derived electric organs varies from gill (*Torpedo*), tail (*Raja, Gnathonemus, Gymnarchus, Gymnotus*), and ocular muscle (*Astroscopus*). Strong electrogenic organs lose the characteristic myofibrils and sarcomeres during transdifferentiation of the organ. In contrast, weakly electrogenic *Gymnarchids *and *Mormyrids *maintain the myofibrillar structures into adulthood [3]. Organs differ in the ability to initiate and propagate an action potential. Generally, marine fish possess organs with electrically inexcitable membranes (lacking voltage-sensitive sodium channels), whereas fresh water fish have organs that are electrically excitable (have voltage-sensitive sodium channels). Succinctly put, the degree of muscle likeness of precursor cells differs among electrogenic fish families. These anatomical differences may represent an evolutionary divergence required for the performance of strong and weak electric organs.

The research presented here focuses on *Torpedo californica *(Pacific electric ray), a cartilaginous fish within the Chondrichthyes class and Torpedinidae family. This species evolved an electric organ capable of generating approximately 45-50 V (electron motive force 110 mV), released in 414 monophasic discharges that last 3-5 ms each, with a total power output up to 1 kW [4-6]. An electrocyte from the electric organ of *Torpedo nobiliana *(Atlantic *Torpedo *with similar length but twice the weight of *T. californica*) measures 5-7 mm in diameter by 10-30 μm thick and 500-1,000 electrocytes are stacked into columns, all with ventrally innervated and dorsally non-innervated membranes aligned [5]. Approximately 50 A of current has been measured from the parallel stacks composing the electric organ of *T. nobiliana*, and about 1 A measured from the series-aligned electrocytes of *Electrophorous *[6]. The postsynaptic membrane of the electric organ in *Torpedo *is rich in nicotinic acetylcholine receptors (AChR) and is multi-innervated with dendrites from four large, heavily myelinated neurons descending from the electric lobe of the brain. The non-innervated membrane is extensively invaginated into structures called caniculi that may be reminiscent of skeletal muscle T tubules [5]. The electrocytes are multinucleated and filled with a gelatinous cytoplasm with an extensive filamentous network. The electrocyte itself has low internal resistance with low resistance across the non-innervated membrane [7]. Insulating septa, extracellular matrix components, blood vessels, nerves, and amoeboid cells have also been described in intercellular regions [8].

Proteins that were originally identified in the *Torpedo *electric organ and subsequently studied in higher vertebrates include agrin, dynein, chloride channel, and rapsyn [9-12]. Also identified in the electric organ are α, β, δ, and γ AChR subunits, α and β dystroglycan, dystrophin, syntrophin, dystrobrevin, receptor tyrosine kinase, tyrosine protein kinase fyn, protein tyrosine kinase fyk, and desmin [13-23]. The electric organ has been used to define the structure and function of creatine kinase and AChR pore [24,25]. These proteins also are characterized at the mammalian neuromuscular junction (NMJ) or are components of skeletal muscle, which is consistent with the *Torpedo *electric organ representing an extreme adaptation of muscle tissue and the NMJ. Thus, the electric organ has served as a model to study the NMJ. However, the number of NMJ proteins described in current mouse, cell culture, and *Drosophila *studies demands a closer look at how the innervated membrane of *Torpedo *electrocytes relates to the NMJ.

From a developmental perspective, *Torpedo *electroblasts are derived from the mesodermal layer that gives rise to branchial arches from which the electric organ and gill musculature form. The primordial electric organ first generates 'muscle-like' cells that are multinucleated and have a single striated myofibril, reminiscent of myotubes in skeletal muscle. At this stage, meromyosin is expressed at high levels and the single striated myofibril has a similar diameter to actin-myosin myofibrilar structures composing sarcomeres [26]. As the electroblast transforms into an electrocyte at the onset of electromotor neuron synaptogenesis, Z-disc-like structures disassemble and degenerate completely [27]. It is thought that the electromotor neuron sends signals that induce the degeneration of the myofibril structures, allowing the elongated cells to flatten into thin electrocytes [28]. Desmin, or a light intermediate filament, replaces the myofibril following disassembly, but keratin, a protein typically associated with epithelium, dominates the intracellular architecture [26,29]. Upon denervation, myofibril-like structures reappear near the synapse but are highly disorganized and short lived [28]. In addition, transcript evidence was shown for myoblast determination protein and myogenic factor 5 expression in adult *Torpedo *electric organ without evidence of protein expression, suggesting strong post-transcriptional regulation of messenger RNA translation and maintenance of a muscle-like programming [30]. No synapse is observed until late phase of electric organ development when the ventral face of electroblasts develop subneural arches that have increased levels of acetylcholinesterase (AChE) and AChRs that reach 300 times the level in skeletal muscle [27,28,30].

From an anatomical perspective, post-transdifferentiation, the electroneuroelectrocyte synapse (electroplate) appears to maintain characteristic synaptic folds and a high density of membrane particles as revealed by electron microscopy and freeze-etch replicas of electric organ tissue [5,31,32]. However, the extensive nerve terminal network, formed by four or five electromotor neurons covers nearly the entire postsynaptic membrane, differs from the minute motor neuron connection with a single mammalian myofiber [5,18].

Despite the electric organ being used as a model for the mammalian NMJ, current literature describes a number of NMJ-associated proteins that have not been characterized in the electric organ. One such protein is low-density lipoprotein receptor-related protein 4 (Lrp4), which forms a complex with muscle-specific tyrosine-protein kinase receptor (MuSK) to facilitate neuronal agrin binding and subsequent initiation of downstream signaling for transcriptional activation of synaptic genes or AChR clustering [33,34]. It is likely that agrin plays a similar role in the electric organ as in the NMJ, transferring communication between the nerve and postsynaptic tissue, but its downstream target, MuSK, is loosely defined. The published sequence for a tyrosine kinase receptor transcript extracted from the *Torpedo *electric organ not only encodes extracellular Ig and frizzled domains and intracellular C-terminal tyrosine kinase domains like human MuSK but also encodes a kringle-like domain that is encoded in proteases and Ror receptor tyrosine kinases [20,35,36]. The orthology of the *Torpedo *tyrosine kinase receptor with mammalian MuSK was demonstrated by inducing AChR clustering in the presence and absence of agrin [37]. Furthermore, the cytoplasmic domain of MuSK binds directly to the tetratricopeptide repeat domain of rapsyn, supporting the presence of MuSK and possibly its downstream effectors in the electric organ [38].

Aside from this knowledge, electrocyte components are undefined mainly because studying the proteome of *T. californica *is limited. A map of its genome does not currently exist to computationally derive a hypothetical protein profile and public databases contain sparse sequence data for this species. While the *Torpedo *genome has not yet been reported, the genome sequence likely would be a relatively blunt instrument to understand the highly specialized structure and function of the *Torpedo *electric organ. For this reason, we sought to understand the molecular components of the electric organ using a combined mRNA (expressed sequence tag (EST)) and proteomics approach.

We have previously reported a preliminary proteome based on two-dimensional matrix-assisted laser desorption/ionization - time of flight/time of flight mass spectrometry (MALDI-TOF/TOF MS) of soluble proteins and shotgun proteomics of insoluble electric organ fractions in which mass spectral mapping was based on a preliminary library composed of 607 cDNA sequences [39]. More recently, we reported sequencing the transcriptome of *T. californica *to assemble a *Torpedo *cDNA library composed of 10,326 sequences assembled into 4,243 non-overlapping contigs [40]. Here, we present a comprehensive electric organ proteome as defined by one-dimensional SDS-PAGE followed by nanospray electrospray ionization quadrupole linear ion-trap tandem mass spectrometry (ESI-LTQ MS/MS) and two-dimensional isoelectric focusing (IEF) SDS-PAGE followed by MALDI-TOF/TOF MS-based approaches of electric organ fractions in which mass spectral mapping was performed using sequences from 10,326 *Torpedo *cDNA sequences and The Universal Protein Resource (UniProtKB/Swiss-Prot). Our results demonstrate concordance between skeletal muscle, NMJ, and electric organ proteomes. In addition, the electric organ expresses several uncharacterized proteins that may function at a synapse.

## Results

### Validation of *Torpedo*-specific protein identification

Tissue fractionation, gel electrophoresis, in-gel tryptic digestion, and mass spectrometry (MS) analysis of the electric organ provided a global proteomic profile comprising 435 proteins (count includes the different subunits, subunit isoforms, isoforms, and types of proteins with unique identifiers and does not include identical proteins found in different spots). Fractionating the electric organ decreases the complexity of its protein constituents and improves detection of low-abundant proteins and protein digestion by decreasing the number of proteins resolved through electrophoresis in a single lane. Confidently identified proteins were determined by a combination of nanospray ESI-LTQ MS/MS spectral searches of our *in silico *translated cDNA library, MALDI TOF/TOF MS and MS/MS spectra from two-dimensional gel spots, and through cross-species spectral matches to UniProtKB/Swiss-Prot amino acid sequences (Additional file 1) [40]. For peptides processed by ESI-LTQ MS/MS and subsequent identification by SEQUEST, our threshold for positive protein identification was two independent peptides, ΔCn >0.1, a variable threshold of Xcorr versus charge state: Xcorr = 1.9 for z = 1, Xcorr = 2.2 for z = 2, and Xcorr = 2.5 for z = 3, protein Xcorr >40, and a peptide probability based score with a *P *value <0.01. For peptides processed by MALDI-TOF/TOF MS and subsequent identification by MASCOT http://www.matrixscience.com/search_form_select.html, our threshold for positive criteria were protein score CI >95%, protein score >69, and proteins with isoelectric points (PI) and molecular weights (MW) that match the gel spot. To represent a concise proteome for the electric organ, all accepted protein identifications were further processed by selecting the highest scoring identification amongst redundant proteins and the removal of lower scoring duplicates. Isoforms and subtypes of proteins were treated as unique identifications and are included in the 435 proteins listed in Additional file 1.

**Figure 1:**
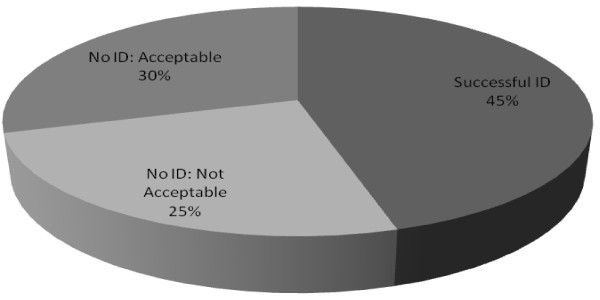
**Identification of *Torpedo *proteins listed in public access databases**. To validate tandem mass spectrometry (MS/MS) data against species-specific sequences, spectra acquired via MS/MS analysis of electric organ fractions were analyzed by the SEQUEST algorithm in BioWorks 3.3.1 software, crossreferencing known and characterized *Torpedo *proteins listed in GenBank. Peptide acceptance criteria was set at ΔCn >0.1, a variable threshold of Xcorr versus charge state: Xcorr = 1.9 for z = 1, Xcorr = 2.2 for z = 2, and Xcorr = 2.5 for z = 3, protein Xcorr >40, and a peptide probability based score with a *P *value <0.01. Protein identifications were compared with a search against UniProtKB (Swiss-Prot and TrEMBL) release 14.0, all species, to maintain consistency with databases used and protein accession numbers reported. Proteins identified are categorized by the likelihood and appropriateness of detection based on protein subcellular location or on the quality of data on public access databases.

As an initial step to validate our data from SDS-PAGE, we performed mass spectral mapping using known *Torpedo *proteins. We created a validation database consisting of all *Torpedo *protein sequences found on public access databases (GenBank and UniProtKB) to search raw spectra. We identified 20 out of 44 *Torpedo *proteins listed on the public access databases (Figure 1, Additional file 2). The *Torpedo *AChR (α, β, γ, δ) and sodium/potassium-transporting ATPase (Na^+^/K^+^-ATPase) subunits (α, β) were amongst the most abundant proteins identified. These two protein complexes are functionally related in that the binding of a ligand to one complex also influences the activity of the other [41]. The Na^+^/K^+^-ATPase is essential for maintaining the electrochemical potential of electrocytes, and activity of this ion exchanger is required for generating electric pulses that *Torpedo *uses for predation. Of the remaining 24 proteins, we do not expect to identify 13 proteins that are expressed in brain, in neurons, or in immune cells. We did not identify 11 listed *Torpedo *proteins that were previously characterized through study of the electric organ (Additional file 2). However, all but one of these sequences are unreviewed by the UniProtKB consortium. In addition, the sequence information for α and β dystroglycan was extremely limited on UniProtKB (a single peptide) and this would, by definition, fall below our two peptide minimum requirement for identification from our MS/MS scans. Another protein, *Torpedo *receptor tyrosine kinase, was translated in UniProtKB/Swiss-Prot from 2 small overlapping ESTs and was in low abundance in the cDNA library previously described (2 in 90,000 clones), suggesting that the very low abundance underlies our inability to identify it by either MS/MS or via our cDNA sequencing [20]. Lastly, dystrophin has multiple isoforms and we identified two of the isoforms (Additional file 2) [42]. Thus, we identified 16 of 17 listed *Torpedo *proteins that have reviewed sequences and are known to be expressed in the electric organ, demonstrating the quality and scope of our data.

Our proteome profile included two uncharacterized open reading frames (ORFs; C1orf123 and C6orf130) and several well characterized mammalian NMJ proteins, including AChR and AChR-associated proteins. Recent publications have characterized new components of the mammalian NMJ and these were also identified in our *Torpedo *electric organ proteome (14-3-3 protein γ, heat shock protein (HSP)90β, HSP 70 kDa protein, laminin subunit α-2, laminin subunit β-2, laminin subunit γ-1, stress-induced phosphoprotein 1, dynamin 1, vesicle-fusing ATPase, Ras-related C3 botulinum toxin substrate 1, prostaglandin E synthase 3, guanine nucleotide-binding protein G(I)/G(S)/G(T) subunit β-1, G subunit β-1, subunit β-2-like1, G(s) subunit α, and Rho GDP-dissociation inhibitor 1, Ras-related protein R-Ras2 (TC21)) [43,44]. Additionally, several presynaptic proteins localized to both synaptic vesicles (synaptic vesicle membrane protein VAT-1, synaptotagmin-B, choline transporter-like protein 1), and the electromotor neuron membrane were identified, showing representation of the presynaptic apparatus of the electric organ.

### *Torpedo *cDNA sequences with both nucleotide and protein sequence similarity to human ORFs

**Figure 2:**
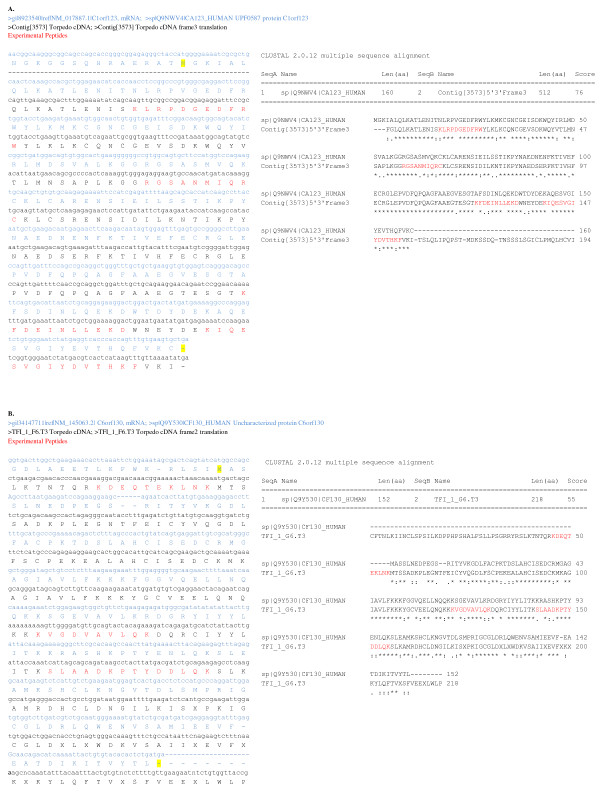
**Sequence alignments between uncharacterized human open reading frames (ORF) and *Torpedo *cDNA**. Two human ORFs were identified by tandem mass spectrometry (MS/MS) analysis of electric organ fractions by the SEQUEST algorithm in BioWorks 3.3.1 software, crossreferencing our in-house *Torpedo californica *cDNA library translated into six reading frames. Comprehensive alignments of nucleotide and protein sequences between uncharacterized human ORFs (blue text) and *Torpedo *cDNA (black text) were compiled from individual ClustalW alignments (default parameters with gonnet matrix) for C1orf123 **(a) **and C6orf130 **(b)**. ClustalW protein alignment is shown separately to highlight protein sequence similarity with the translated cDNA sequence (Expasy translate tool) and peptides identified by mass spectral mapping (highlighted in red). Start and stop amino acids are highlighted in yellow.

Electric organ peptides mapped to the uncharacterized human ORFs C1orf123 and C6orf130, which aligned with high sequence similarity to *Torpedo *cDNA sequences, supporting their expression in the electric organ. Human nucleotide and protein sequences were obtained from GenBank for alignment with translated *Torpedo *cDNA sequence (Expasy translate tool) using EBI ClustalW (default parameters with gonnet matrix). Sequence alignments between human ORF nucleotide and protein sequences and *Torpedo *cDNA nucleotide and translated nucleotide sequences are shown in Figure 2. In all, 79% of amino acids in the translated *Torpedo *cDNA sequence Contig [3573] are identical to C1orf123 and 59% of TF_1_F6.T3 with C6orf130. TF_1_F6.T3 cDNA sequence is from a single insert and not a contig such that coverage may be reduced by sequencing errors that were not corrected for by a consensus of multiple reads like Contig [3573]. However, peptide SLAADKPTYDDLQK is unique to C6orf130 when queried in blastp (word size 2, PAM30 matrix, *Homo sapiens*) supporting the identification of this ORF. These ORFs demonstrate that further investigation of the electric organ transcriptome may advance our knowledge of the human proteome.

### Global proteomic profile classified according to UniProtKB/Swiss-Prot annotation

**Figure 3:**
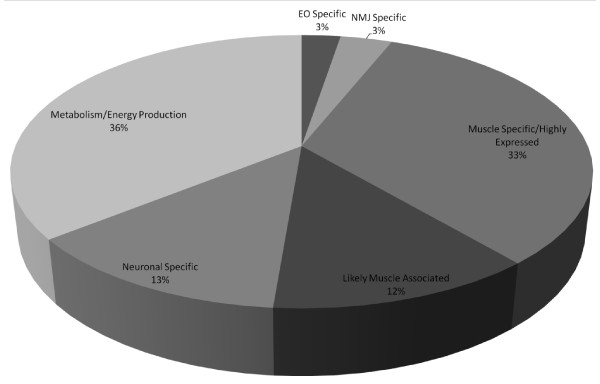
**Classification of proteins identified in electric organ fractions by tissue association or function as determined by UniProtKB annotation**. Electric organ fractions were separated one dimensionally and analyzed by nanospray electrospray ionization quadrupole linear ion-trap tandem mass spectrometry (ESI-LTQ MS/MS). Mass spectral matching of raw spectra against UniProtKB and *Torpedo *cDNA library was performed in BioWorks 3.3.1 in which the peptide acceptance criteria was set at ΔCn >0.1, a variable threshold of Xcorr versus charge state: Xcorr = 1.9 for z = 1, Xcorr = 2.2 for z = 2, and Xcorr = 2.5 for z = 3, protein Xcorr >40, and a peptide probability based score with a *P *value <0.01. All cDNA sequences were queried in blastx (standard genetic code, Swiss-Prot, default algorithm parameters except for BLOSSUM80 scoring matrix) for identification via sequence similarity with a known protein, first across all species and then against *Homo sapiens *selected database. Cytosolic proteins were separated two dimensionally, analyzed via matrix-assisted laser desorption/ionization - time of flight/time of flight mass spectrometry (MALDI-TOF/TOF MS), and identified by MASCOT. Identification criteria was set at a protein score CI >95%, protein score >69, and proteins with isoelectric points (PI) and molecular weights (MW) that match the gel spot. Each identification was queried in UniProtKB for annotation of tissue expression and or function then categorized by the sections composing the pie chart. (See Additional file 3 for a list of proteins composing the pie chart.)

To obtain a preliminary identification for each *Torpedo *cDNA sequence identified in the spectral data, all sequences were queried in blastx (Swiss-Prot sequence, word size 3, BLOSSUM80 matrix) across all species and then against human (See Additional file 1 for a full list of cDNA sequences with blastx results). Only the top ranking aligned sequence was accepted for identification of the cDNA sequence. The blastx identification allowed cDNA sequences to be grouped with the UniProtKB list of identifications for classifying the proteins as NMJ, muscle, likely in muscle, and metabolic proteins according to UniProtKB/Swiss-Prot annotation (Figure 3 andAdditional file 3). A total of 33% of proteins are known muscle proteins, 3% of which are located at the NMJ. A total of 36% are involved in metabolism and 3% are known to be electric organ specific. Ingenuity Pathway Analysis (IPA version 8.8-3204) of all UniProtKB and *Torpedo *cDNA identifications classified 40 molecules (*P *value 2.93E-09 to 1.18E-02) involved in skeletal and muscular system development and function, the top physiological system designated from our list of identifications.

**Figure 4:**
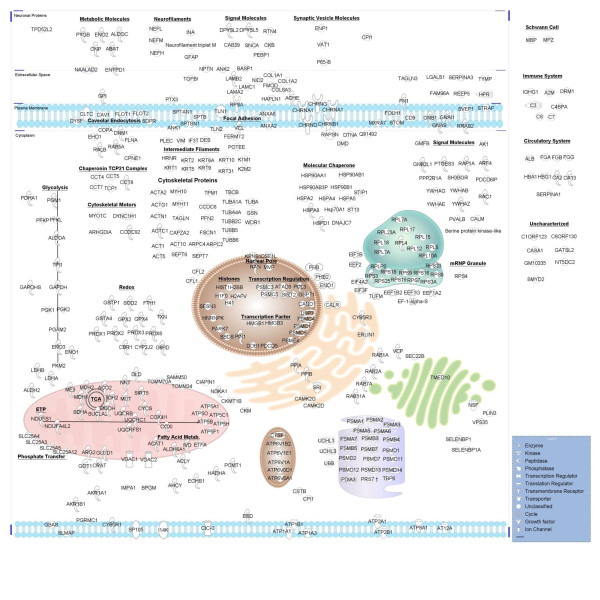
**Virtual *Torpedo *electrocyte**. All identifications from UniProtKB/Swiss-Prot and *Torpedo *cDNA searches of fractions analyzed by nanospray electrospray ionization quadrupole linear ion-trap tandem mass spectrometry (ESI-LTQ MS/MS) and matrix-assisted laser desorption/ionization - time of flight/time of flight mass spectrometry (MALDI-TOF/TOF MS) were mapped to cellular regions based on UniProtKB annotations using the Path Designer tool in Ingenuity IPA 8.8-3204.

**Figure 5:**
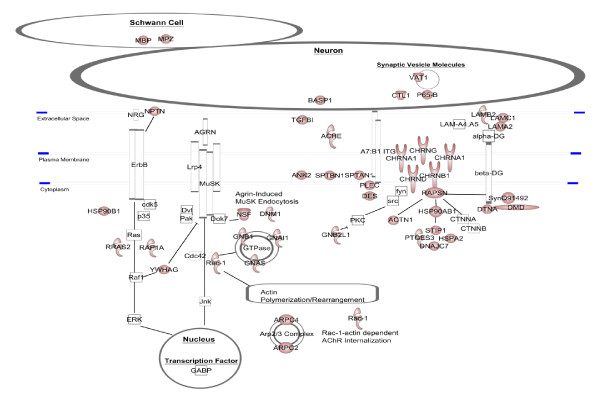
***Torpedo *electrocyte proteins in context of the mammalian neuromuscular junction (NMJ)**. Electric organ identifications are displayed in context of the mammalian NMJ paradigm. Red shapes indicate proteins we identified in our *Torpedo *electric organ proteome. White shapes are proteins we did not identify. This image was created using Path Designer in Ingenuity IPA 8.8-3204.

To summarize the electric organ proteome, we used IPA Path Designer tool to map the annotated subcellular location of each protein identified (Figure 4). This also provides a virtual model of the electrocyte to assess how it may relate to skeletal muscle and the NMJ. The virtual electrocyte revealed several proteins believed to be muscle specific or highly abundant in muscle, confirming the muscle-like identity of the organ (Additional file 3). It also depicted relatively intact pathways for energy metabolism (oxidative phosphorylation and glycolysis), protein processing (translation initiation, elongation, trafficking, and proteasome degradation) and several components involved in redox reactions and caveolar endocytosis. A prominent feature is an abundance of cytoskeletal proteins to include molecular motors, capping and folding proteins, and focal adhesion molecules. Also notable are a number of proteins that interact with known NMJ proteins (Figure 5). Finally, the virtual electrocyte reveals several relatively uncharacterized proteins such as membrane proteins receptor expression-enhancing protein 5, MIP18 family protein FAM96A, WD repeat-containing protein 1, and matrix-remodeling-associated protein 7.

### Electric organ proteome compared to skeletal muscle proteome to assess the degree of 'muscle likeness'

**Figure 6:**
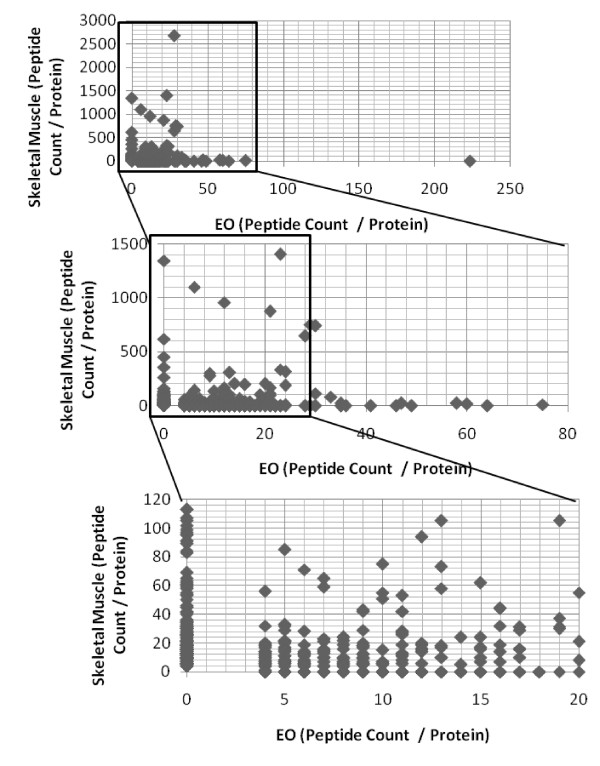
**Electric organ proteome overlaps with mouse skeletal muscle proteome but shows tissue-specific protein expression**. Mouse skeletal muscle (tibialis anterior muscle or gastrocnemius muscle) and the *Torpedo *electric organ were fractionated and processed under similar conditions as stated under Figure 1. Mouse skeletal muscle proteins were identified by BioWorks 3.3.1 referencing only UniProtKB/Swiss-Prot. Electric organ (EO) and skeletal muscle proteins were compared and graphed in Microsoft Excel 2007 based on the number of peptides per protein identified in each tissue. EO proteins are mapped (#peptides/protein) on the × axis and mouse skeletal muscle on the y axis. The lower two graphs represent zoomed sections for visual clarity.

Electric organ literature claims that a 'muscle-like' phenotype is maintained after transdifferentiation. In our profile, several proteins are considered highly expressed in skeletal muscle or are muscle specific to include AChR subunits α,β,δ, and γ, rapsyn, syntrophin, L-lactate dehydrogenase A chain, phosphoglycerate mutase 2, creatine kinase M-type, cofilin 2, sorcin, 14-3-3 protein γ, myosin 11, actin, aortic smooth muscle, transgelin, dystrophin, dystrobrevin α, desmin, plectin 1, HSP90β, laminin subunit β-2, and SR Ca(^2+^)-ATPase 1. As a further step to compare the skeletal muscle versus the electric organ repertoire of proteins, we compared the proteins identified in the electric organ presented in this paper to a mouse skeletal muscle proteome produced in our laboratory using similar methods. Plotting the number of peptides for each protein composing the electric organ or skeletal muscle proteome not only visually displays the overlap in proteins in both tissues but more importantly displays the detectable proteins unique to each tissue, those lying on the × and y axis corresponding to tissue type (Figure 6). Analysis showed the distribution of these proteins differed in biological processes and molecular function (Table 1). Proteins composing the myofibrillar apparatus or are involved in calcium transport are present in the skeletal muscle proteome and absent in the electric organ proteome, as expected given that the electrocytes are non-contractile cells. However, no common NMJ proteins were identified in the skeletal muscle proteome but are amongst the highest expressed proteins in the electric organ. This was also expected for an analysis based on total muscle extract given the limited size and number of endplates in skeletal muscle.

**Table 1:** *T. californica *electric organ proteome shows tissue-specific proteins when compared to mouse skeletal muscle proteome

Unique to skeletal muscle proteome	Unique to electric organ proteome
Development (myogenesis):	Myofibrillar:

UN45B	Cytoskeleton, actin M-band

Myofibrillar:	ANK1

Cytoskeleton, actin	Light chain part of A-band

TITIN	MYL9

Z-disk	Neuromuscular junction (NMJ):

ACTN2	ACES

ACTN3	ACHA

MYOTI	ACHB

PP2BA	ACHD

Class II myosins (A-band)	ACHG

MYBPH	DTNA

MYH1	HSP70

MYH3	HSP90B

MYH4	RAPSN

MYH6	NMJ-ECM

MYH7	LAMA2

MYH8	LAMB2

MYPC2	LAMC1

Light chain part of A-band	Cytoskeleton:

MYL1	Cytoskeleton, actin

M-band	SEPT6

MYOM1	sept7

OBSCN	ACTG

Contraction	ADDG

PHKG1	ACTC

MYLK2	ANK2

TNNC2	ARPC2

TNNI2	ARPC4

TNNT3	CCDC6

TPM2	COF1

ACTN4	COF2

Cytoskeleton:	DNJC7

Cytoskeleton, actin	FERM2

ACTS	PROF2

ML12B	SLMAP

MLRS	TCPA

MLRV	TLN2

Actin capping-binding	Actin capping-binding

CAPZB	CAZA2

RADI	SPTB1

XIRP1	Cytoskeleton, microtubule

Cytoskeleton, microtubule	DYN1

CLIP1	SIRT2

KINH	TBB1

PBIP1	TBB2

STIM1	TBB5

TBB2A	Intermediate filaments

TBB2C	K1C9

Sarcoplasmic reticulum/calcium pathways:	K1H1

AT2A2	NFH

AT2A3	NFL

CALU	NFM

JPH2	Sarcoplasmic reticulum/calcium pathways:

SRCA	SORCN

CASQ1	Ion channels:

CASQ2	Chloride channel

KPB1	CICH

Ion channels:	Calcium ATPase

Voltage-sensitive calcium channels	AT2B1

CA2D1	AT8A1

CAC1S	Hydrogen-potassium ATPase

CACB1	AT12A

Potassium channel	AT1A

TM38A	Sodium-potassium ATPase

Sodium-potassium ATPase	AT1A3

AT1A2	AT1B1

AT1B2	Extracellular matrix (ECM):

Oxygenation (muscle):	NID2

MYG	FINC

ECM:	CO1A1

CO6A1	CO1A2

COEA1	CO6A3

ITB1	BGH3

NID1	HPLN1

PEPD	Neuronal:

PGS2	AINX

Neurogenesis:	VAMP3

NDKA	

NDRG2	

Proteins on the × and y axis of Figure 6, indicating unique identification in the corresponding tissue, were listed based on biological process, molecular function, and cellular localization. See Additional File 1 for expanded names of all abbreviated proteins.

## Discussion

### *T. californica *proteome and defining the NMJ proteome for accurate comparison

Our goal was to generate a proteomic profile of the *T. californica *electric organ, both to assess its similarity to the mammalian NMJ proteome and to provide novel candidate proteins for localization to the NMJ. We and others have carried out microdissection of the NMJ region and messenger RNA profiling to characterize the NMJ constituents, but these have proven technically challenging and have fallen short in describing a broader proteome [45,46].

A key resource for our one-dimensional ESI-LTQ MS/MS and two-dimensional MALDI-TOF/TOF MS profiles were cDNA sequences from the electric organ that enabled mass spectral mapping [40]. Of 435 proteins we identified in the electric organ, 300 (69%) showed ≥2 peptides that mapped to our *Torpedo *cDNA sequences while the remaining 135 (31%) were characterized via cross-species peptide spectral mapping to mammalian proteins. We found that 48% of identified proteins were highly expressed in skeletal muscle or are muscle specific, which supports the 'muscle-like' lineage of the electric organ. The proteome includes cytoskeletal, glycolytic, translational, and degradative proteins. The high prevalence of glycolytic enzymes likely is necessary to support the high metabolic load of the organ that is required for establishing and maintaining the membrane potential. The abundance of proteasome and degradative enzymes is in line with high protein turnover and degradation during synapse renewal as well as transdifferentiation from muscle precursor cells into the electric organ. Additionally, we identified several proteins that are expressed by non-electrocyte cells composing the electric organ, such as the electromotor neuron proteins, Schwann cell proteins, and proteins of the immune and circulatory systems.

To compare our *Torpedo *data to previous studies of the mammalian NMJ, we scanned the literature for known NMJ proteins, grouped these into three categories, and overlaid our *Torpedo *proteome with these groups. The first category was limited to proteins in which experimental knockout (loss of function) data suggested an important functional role in postsynaptic architecture and function (for example, disruption of morphology) (Additional file 4; see also references cited therein). The second category included protein-protein networks nucleated by the key functional candidates in category 1. Most category 2 proteins were shown to attenuate AChR clusters when mutated, inhibited, or deleted. Finally, the remaining category contained proteins strictly concentrated at the endplate but do not alter AChR clusters or synapse morphology (category 3). We identified rapsyn, β-spectrin, Ras-related C3 botulinum toxin substrate 1, and laminin subunit β-2 from category 1, HSP90β, HSP 70 kDa protein, α syntrophin, 14-3-3 protein γ, dynamin, vesicle-fusing ATPase, α-actinin, utrophin, and Ras from category 2, and ankyrin, desmin, and dystrobrevin from category 3 (16/38 molecules listed). The presence of these molecules suggests the neuromuscular protein machinery supporting the cholinergic endplate coincides with the electric organ and may serve as a model NMJ to study these proteins.

In addition to the few *Torpedo *proteins characterized at the cholinergic synapse (AChR subunits α, β, δ, and γ, ACHE, rapsyn, 14-3-3 γ, syntrophin) we identified several uncharacterized proteins in the electric organ known to play a role in maintaining AChR clustering and in transducing signals between the membrane and nucleus (Figure 5). These proteins include laminin subunits α-2, β-2, and γ-1, HSP90β, HSP 70 kDa protein, stress-induced-phosphoprotein 1, dynamin 1 and vesicle-fusing ATPase, α-actinin, prostaglandin E synthase 3, Ras-related C3 botulinum toxin substrate 1, guanine nucleotide-binding protein G, guanine nucleotide-binding protein subunit β-2-like1, Rho GDP-dissociation inhibitor 1, and Ras-related protein R-Ras2. Below, we describe each of these electric organ components as they relate to the mammalian NMJ.

### *T. californica *proteome related to AChR clustering

A key event in the formation of the neuromuscular junction is the clustering of AChRs to focal points underlying motor neuron synapses. At the developing synapse, a key protein complex involved in clustering is the laminins: multisubunit glycoprotein complexes consisting of α, β, and γ chains, each with multiple isoforms, assembled in a trimer of equal stoichiometry. Laminin subunits α2, β2, and γ1 are seen most frequently in mature NMJs where they form the laminin 4 complex (also called S-merosin); we identified each of these subunits in the *T. californica *proteome. Subunit γ1 facilitates the interaction between AChR and α7β1 integrins to prime cluster formation prior to neuronal agrin release or when agrin levels are low [47,48]. At the mature synapse, the laminin complex interacts with extracellular matrix (integrins and agrin) and postsynaptic membrane components (basal cell adhesion molecule (Bcam), α dystroglycan, and AChR) to link the extracellular regions with the intracellular cytoskeleton and to regulate the release of intracellular calcium directed at AChR cluster formation [48-50]. Subunit β2 also assists in the development of synaptic folds and Schwann cell placement at the synapse [51]. The laminin receptors characterized at the synapse, Bcam and dystroglycan, were not identified but dystroglycan was previously characterized in the electric organ [13,52]. However, we identified laminin receptor 1 (LamR1 or RPSA), a known binding partner for the laminin complex in the electric organ (α2, β2, and γ1; also called S-merosin or laminin 2/4). Interestingly, LamR1 has not been previously reported at the NMJ [53,54].

Recent evidence supports the role of HSP90β and HSP 70 kDa protein (HSP70) as stabilizing chaperones of NMJ proteins. HSP90β was shown to interact directly with rapsyn at its tetratricopeptide repeat (TPR) domain following its binding to surface AChR clusters. Recruitment of HSP90β is believed to stabilize AChR-rapsyn binding to influence AChR stability and maintenance and also may associate with α dystrobrevin and α syntrophin [44]. HSP70 may be a cochaperone of HSP90β along with DnaJ homolog subfamily C member 7, HSP40, and prostaglandin E synthase 3 (p23). p23 is involved in stabilizing the ATP-bound conformation of HSP90, permitting the release of activated interacting partners [55]. We identified p23 in our *Torpedo *cDNA library, suggesting its role as a cochaperone with HSP90 in the electric organ. We also identified stress-induced phosphoprotein 1, which facilitates the interaction between HSP90β and HSP70 [56].

### Intracellular signal transduction

We identified proteins (vesicle-fusing ATPase (NSF), dynamin, Ras-related C3 botulinum toxin substrate 1 (Rac1), G proteins) involved in agrin-dependent MuSK activation and subsequent AChR clustering and synaptic gene transcription (Figure 5). In this process, agrin binds Lrp4 to activate MuSK and its subsequent internalization via clathrin-mediated endocytosis and to activate expression of MuSK interacting proteins. Dynamin 1 and NSF are involved in receptor-mediated endocytosis, vesicle transport, and protein trafficking. NSF is essential for agrin-induced receptor-mediated endocytosis of MuSK and activation of its downstream signaling molecules Abl kinase and Rac1 in C2C12 cells, which promote AChR clustering [57]. In addition, dynamin supports clathrin-coated vesicles formed upon agrin-induced endocytosis of MuSK, which is translocated into lipid rafts for activation and signaling.

Several studies support the 'signaling endosome hypothesis' in which neurotrophic factors initiate ligand-mediated endocytosis of receptor tyrosine kinases into clathrin-coated vesicles that contain activators such as G proteins and downstream effector molecules involved in Ras-mitogen-activated protein kinase (MAPK) signaling [58-61]. In the electric organ, we identified guanine nucleotide binding proteins and inhibitors that act on Rho family of Ras-related G proteins that may be involved in signaling endosomes. These include G subunit β-1 (GNB1), subunit β-2-like1 (RACK1), G(s) subunit α (GNAS1), and Rho GDP-dissociation inhibitor 1 (ARHGDIA). GNB1 composes part of the catalytic machinery of GTPases and provides docking regions for interacting proteins. ARHGDIA prevents the release of GDP from Rho proteins (Rho, Rac, cdc42, TC10). RACK1 is the receptor of protein kinase C (PKC), which is known to inactivate Rho; PKC also phosphorylates serine residues of AChR δ subunit to promote receptor desensitization and disassembly [62-64]. Identification of several proteins involved in ligand-mediated endocytosis, activators and inhibitors of GTPases, signaling, lysosomal and proteasomal degradation support the maintenance of protein function across myogenic-derived cell types.

Interestingly, Rac1 is involved in clathrin/dynamin-independent endocytosis of AChR following binding with bungarotoxin [65]. Rac1 functions in actin polymer rearrangement to create compartments for AChR surface sequestration and, most likely, polymerization of the cytoskeletal network involved in vesicle transport to the lysosome for degradation. Rac is a key mediator of receptor surface sequestration in addition to its role in actin polymerization and rearrangement, which controls the number and arrangement of receptors at the synapse to modulate synaptic transmission.

Our proteome also includes an inhibitory protein of synaptic gene expression. The 14-3-3 γ (YWHAG), extracted from *Torpedo *electric organ, reduced the expression of MuSK, AChR subunits ε and α, utrophin, and rapsyn and resulted in aberrant NMJ morphology [43]. It is known that 14-3-3 γ interacts with the N-terminus of Raf-1, HSP90 interacts with the C-terminus of Raf-1, and Ras (RRAS2 (TC21)) binds to the Raf-1-HSP90-p50 complex, causing the complex to translocate to the plasma membrane and become an active kinase for phosphorylating mitogen-activated protein kinase kinase (MEK) [66-68]. PKC also is a target of 14-3-3 γ.

### *T. californica *proteome: limits as a NMJ model

Several critical NMJ proteins are absent from our data. Most notable are proteins within the two major networks responsible for postsynaptic stabilization and gene expression. The first network is the Agrin-MuSK-Lrp4/Src/Rapsyn network involved in AChR cluster formation and stabilization (Figure 5). The second is the Agrin-MuSK/NRG-ErbB/MAPK/GABP network for transcriptional activation of synaptic genes (Figure 5). Absent molecules involved in these pathways include downstream of tyrosine kinase 7 (Dok7), dishevelled (Dvl), PAK, RAF-1, and extracellular signal-regulated kinase (ERK). In addition, several *Torpedo *proteins with UniProtKB/TrEMBL annotation that are expressed at the NMJ were not detected by tandem mass spectrometry analysis of subcellular fractions. These include α and β dystroglycan homologs, the receptor tyrosine kinase similar to MuSK, and protein tyrosine kinases Fyn and Fyk. However, we did identify dystrophin, dystrobrevin, and syntrophin that compose the dystroglycan complex and we did show several molecules that may be up and downstream of receptor tyrosine-protein kinase ErbB (neuroplastin (NPTN), Ras-related protein R-Ras2 (RRAS2) or Ras-related protein Rap-1A (RAP1A), HSP90β).

We failed to identify relatively well characterized mammalian NMJ proteins in our survey, including Lrp4, MuSK, Dok7, Src and Fyn Kinase, Dvl, ErbB2, PKC (category 1), and agrin, laminin subunits α4, α5, PAK1, Rho, cyclin-dependent kinase 5 (cdk5), ephexin1, neuregulin, ETS transcription factor, Raf, MEK, MKK4, c-Jun N-terminal kinase (JNK), and c-Jun (category 2). This may reflect technical issues with the sensitivity of our proteomics methods and parameters (for example, false negative and low maximum mass range for glycosylated peptides), challenges in mapping peptide spectral data to the partial cDNA sequence coverage or to cross-species transcript units, or significant differences in the structure and function of the electric organ compared to the mammalian NMJ. Our study is based on non-targeted proteomics and it may be possible to identify these specific proteins in the electric organ using a more targeted approach. The literature on the *Torpedo *electric organ supports the presence of agrin, α and β dystroglycan, MuSK, and Src kinases, which strengthens the organ's use as a model NMJ.

## Conclusions

The virtual electrocyte revealed that the *Torpedo *electric organ is a resource for several uncharacterized proteins whose function may be clarified in future studies. Knockout and reporter assays of C6orf130, C1orf123, matrix-remodeling-associated protein 7, protein NipSnap homolog 2, septin-6, prohibitin 2, GATS-like protein 2, SH3 domain-binding glutamic acid-rich protein, and 14-3-3 protein ζ/δ in mouse skeletal muscle may clarify their subcellular roles, which may reveal novel components involved in AChR expression and maintenance. The electric organ will continue to serve as a model of membrane excitability and electrogenesis as it is abundant in AChR and the Na^+^/K^+^-ATPase channels and may be used as a model to design a prototype biobattery.

Based on our identification of electric organ proteins that match proteins in the three categories of our defined 'NMJ proteome' and the persistent similarity to skeletal muscle, the electric organ can serve as a repository of these NMJ molecules that are in low abundance in skeletal muscle. However, the absence of several NMJ components involved in synaptic gene expression and AChR clustering in our model limits our ability to conclude that it indeed represents the mammalian NMJ that is maintained similarly. This study offers a more detailed understanding of the electrocyte protein repertoire with insight into the presence and absence of proteins between these two related tissues. It reflects their unique tissue-function specializations and insight into evolutionary conservation and divergence between synaptic gene expression, maintenance, and regulation. The data raises questions whether the pathways responsible for AChR clustering are required in the electrocyte given its dense innervations and high AChR expression or whether electromotor neurons support the postsynapse with different neurotrophic or signaling molecules than mammalian motor neurons such that the neuregulin-ErbB pathway is unnecessary.

## Methods

### Sequencing and mass spectral database indexing of *T. californica *cDNA library

The 10,326 cDNA sequences utilized for proteomics mass spectral mapping database have been previously described [40]. All *T. californica *sequences were saved as a Fasta database and indexed in BioWorks 3.3.1 SP1 (Thermo Fisher Scientific, Waltham, MA) as trypsin digested protein sequences from the translation across all six reading frames.

### Fractionation of the electric organ

*T. californica *electric organ was fractionated by grinding and homogenizing electroplax in lysis buffer (0.25 M sucrose, 20 mM Tris pH 8.0, 25 mM KCl, 5 mM MgCl_2_, Roche Mini Complete Protease Inhibitor and PhosStop Phosphatase Inhibitor (Roche, Branchburg, NJ, USA) [69]. Tissue homogenate was centrifuged at 627 *g *(2,500 rpm) for 15 min at 4°C. The pellet (P1) was saved for further purification and the supernatant was centrifuged at 10,000 *g *for 20 min at 4°C. The pellet (P2) was saved for further purification and the supernatant was ultracentrifuged at 100,000 *g *for 60 min at 4°C resulting in pellet P3 and supernatant S3.

P1 was processed further by homogenizing the isolated pellet in 2-3 ml lysis buffer. The homogenate was filtered through a 100 μm nylon filter (BD, Franklin Lakes, NJ, USA) to remove connective tissue. The filtrate was centrifuged at 627 *g *for 15 min at 4°C. This pellet was resuspended in 2 M STM buffer (2 M sucrose, 50 mM Tris-HCl pH 8.0, 5 mM MgCl_2_, Roche Mini Complete Protease Inhibitor and PhosStop Phosphatase Inhibitor) and placed in an ultracentrifuge at 80,000 *g *for 35 min at 4°C. The resultant pellet was resuspended in EBC buffer (50 mM Tris-HCl pH 8.0, 120 mM NaCl, 1% Triton-X 100, Roche Mini Complete Protease Inhibitor and PhosStop Phosphatase Inhibitor). After 15 min incubation at 4°C, the suspension was passed through a 20-gauge needle ten times to lyse any cells. Soluble (S1) and insoluble fractions were separated by centrifugation at 9,000 *g *for 30 min at 4°C. The pellet (P1.1) was resuspended in EBC buffer.

P2 was resuspended in 0.5 ml HDP buffer (10 mM 4-(2-hydroxyethyl)-1-piperazineethanesulfonic acid (HEPES), 1 mM dithiothreitol (DTT), Roche Mini Complete Protease Inhibitor and PhosStop Phosphatase Inhibitor). After 30 min incubation on ice, the suspension was sonicated (Sonifier Cell Distributer 350, Branson Scientific Danbury, CT) on ice for five initial pulses, paused for 30 s, then a final ten pulses (50% Duty Cycle, Pulsed-Hold, Output Control Limit 3). The lysate was centrifuged at 9,000 *g *for 30 min at 4°C. The supernatant was saved as S2. P2.1 was resuspended in ME buffer (20 mM Tris-HCl, 0.4 M NaCl, 15% glycerol, 1 mM DTT, 1.5% TritonX-100, Roche Mini Complete Protease Inhibitor and PhosStop Phosphatase Inhibitor), incubated for 30 min at 4°C with rocking, then centrifuged at 9,000 *g *for 30 min at 4°C. The supernatant of solublized P2.1 was saved as P2.1.

S1 and S3 fractions were concentrated in a speed vacuum. P3 was suspended in EBC buffer. Each fraction except P2.1 and S2 was desalted by passing the sample through a BioSpin6 column before protein quantitation using the DC Protein Assay (BioRad, Hercules, CA, USA). Protein extracts were stored at 80°C until electrophoresis.

### Protein isolation and identification

#### Protein separation

Proteins within each fraction were resolved using one-dimensional SDS-PAGE on Novex NuPage^® ^3% to 8% Tris-Acetate MidiGel and 4% to 12% Bis-Tris MiniGel Systems (Invitrogen, Carlsbad, CA, USA) according to manufacturer's directions such that 2.5-400 kDa proteins may be isolated and prepared for ESI-MS/MS analysis. In addition, 250 μg of cytosolic proteins were resolved by two-dimensional electrophoresis as previously described with minor differences [70]. The immobilized pH gradient (IPG) strip was rehydrated for 12 h at 20°C and was focused at 20°C using the following conditions: 250 V for 15 min, 8,000 V for 2.5 h, 500 V hold. Following isoelectric focusing (IEF), the IPG strip was incubated in equilibration buffer (6 M urea, 50 mM Trizma preset crystals (pH 8.8), 2% SDS (w/v), 30% glycerol (w/v), 0.002% bromophenol blue) with 1% DTT for 20 min followed by a 20 min incubation in equilibration buffer with 2% iodoacetamide. Each gel was fixed for 30 min in 5% acetic acid, 45% methanol solution, stained with Bio-Safe Coomassie (Bio-Rad) for 60 min, and destained in distilled water overnight.

#### Protein digestion

Multiple molecular weight bands and spots were manually excised from the gel (Additional file 5) and processed for in-gel digestion with 12.5 ng/μl Trypsin Gold (reconstituted according to manufacturer's directions, Promega, Madison, WI, USA) in 50 mM NH_4_HCO_3 _as previously described [71].

#### Protein identification: ESI-LTQ-MS/MS

Recovered peptides from SDS-PAGE were analyzed using nanospray ESI-LTQ MS/MS as previously described, with minor differences [72]. Peptides were loaded onto a C18 reverse-phase column for 10 min at a flow rate of 5 nl/min then separated at a flow rate of 250 nl/min. A 65 min linear gradient eluted peptides. The LTQ operated in data-dependent mode to perform one full MS scan (300-2,000 m/z) to select the five most intense peaks through dynamic exclusion for MS/MS analysis via collision-induced dissociation (CID) with helium at 35% normalized energy. Raw spectra were analyzed by the SEQUEST algorithm in BioWorks 3.3.1 software, crossreferencing our *T. californica *cDNA library translated into six reading frames and The Universal Protein Resource (UniProtKB/Swiss-Prot) release 14.0 [73]. Peptide acceptance criteria was set at ΔCn >0.1, a variable threshold of Xcorr versus charge state: Xcorr = 1.9 for z = 1, Xcorr = 2.2 for z = 2, and Xcorr = 2.5 for z = 3, protein Xcorr >40, and a peptide probability based score with a *P *value <0.01. Spectral data (.raw files) were first converted into MS2 file format (.ms2 files) using pXtract, default settings, and then into PRIDE XML format using PRIDE Converter for upload onto the PRIDE database [74-76]. Data can be found under the project name '*Torpedo californica *Electric Organ Proteome', accession numbers: 16,474-16,476.

#### Protein identification: MALDI-TOF/TOF MS

Two-dimensional IEF SDS-PAGE separated cytosolic peptides were processed and analyzed for protein identification as previously described with the following additional details. Data was acquired using the following parameters: mass range 500-4,000 Da, minimum S/N 20, mass tolerance ± 2 m/z, minimum peak match 4, maximum outlier error 10 ppm, monoisotopic mass [70]. MS and MS/MS spectra of peptides were searched against the UniProtKB/Swiss-Prot (release 15.0) by MASCOT using the following parameters: MS peak filtering mass range 800-4,000 Da, minimum S/N 10, peak density filter 50 per 200 Da, maximum number peaks 65; MS/MS peak filtering: mass 60 Da to 20 Da below precursor mass, lowest precursor 707.46 Da, peak density filter 50 per 200 Da, maximum number peaks 65, fixed modification carbamidomethyl (C), variable modification oxidation (M), fragment ion tolerance 0.3, precursor tolerance 0.5. Proteins identified by the MASCOT algorithm were filtered based on proteins identified with MS/MS spectra, protein score CI >95%, protein score >69, proteins with PI and MW that match the gel spot. All .dat files of spectral data were also uploaded to the PRIDE database under the same project title as ESI-LTQ-MS/MS data stated above.

#### Lipid raft assay of fraction

To isolate membrane proteins localized to lipid rafts, membrane was isolated from 3 g of electric organ according to the above procedure (protein separation) with minor modifications. Tissue homogenate was centrifuged twice at 627 *g *for 15 min at 4°C and the supernatant passed through a 40 μm filter to clear cellular debris. The supernatant was ultracentrifuged at 100,000 *g *for 60 min at 4°C to collect an insoluble pellet rich in membrane proteins. Lipid rafts were isolated from electric organ membrane fraction following previously published guidelines with the following modifications: the gradient was ultracentrifuged at 100,000 *g *[77]. Visible bands were collected and centrifuged at 14,000 *g *for 30 min. The resultant pellets were resuspended in EBC buffer. Intermediate solutions were also collected and concentrated by vacuum centrifugation. All collected fractions were subjected to one-dimensional SDS-PAGE on Novex NuPAGE^® ^4% to 12% Bis-Tris MiniGel using NuPAGE^® ^MES SDS Running Buffer (Invitrogen, Carlsbad, CA) according to the manufacturer's instructions. Protein bands were manually excised and processed for ESI-LTQ-MS/MS analysis as described earlier.

## Competing interests

The authors declare that they have no competing interests.

## Authors' contributions

SEM carried out the proteomic profiling of the *Torpedo californica *electric organ, data comparison with mouse skeletal muscle proteome, the development of images projecting the electrocyte proteome as well as the proteome within the context of the NMJ, and preparation of the manuscript. KJB helped design and execute proteomic profiling and data analysis as well as assisting in the write-up of methodology and manuscript editing. EPH provided the conceptual design of the project and insight into data analysis as well as drafting of the manuscript. All authors have read and approved this manuscript. SEM is a predoctoral student in the Biochemistry and Molecular Genetics Program of the Institute for Biomedical Sciences at the George Washington University. This work is from a dissertation to be presented to the above program in partial fulfillment of the requirements for the PhD degree.

## Supplementary Material

Additional file 1 ***Torpedo californica *electric organ proteome**. All 435 proteins, identified across different sample processing and mass spectral data acquisition techniques, representing the *T. californica *proteome are listed in separated tabs based on the method of identification. Electric organ fractions were separated by SDS-PAGE and analyzed by nanospray electrospray ionization quadrupole linear ion-trap tandem mass spectrometry (ESI-LTQ MS/MS) or matrix-assisted laser desorption/ionization - time of flight/time of flight mass spectrometry (MALDI-TOF/TOF MS). For ESI-LTQ MS/MS, mass spectral matching of raw spectra against UniProtKB and *Torpedo *cDNA library was performed in BioWorks 3.3.1 in which the peptide acceptance criteria was set at ΔCn >0.1, a variable threshold of Xcorr versus charge state: Xcorr = 1.9 for z = 1, Xcorr = 2.2 for z = 2, and Xcorr = 2.5 for z = 3, protein Xcorr >40, and a peptide probability based score with a *P *value <0.01. All cDNA sequences were queried in blastx (standard genetic code, Swiss-Prot, default algorithm parameters except for BLOSSUM80 scoring matrix) for identification via sequence similarity with a known protein, first across all species and then against *Homo sapiens *selected database (column C: all species/*Homo sapiens*). Score of blastx alignments: black ≥200, pink = 80-200, green = 50-80. For MALDI-TOF/TOF MS of cytosolic proteins resolved two dimensionally, acquired data were searched against UniProtKB/Swiss-Prot (release 15.0) by MASCOT using the following parameters: MS peak filtering mass range 800-4,000 Da, minimum S/N 10, peak density filter 50 per 200 Da, maximum number peaks 65; MS/MS peak filtering: mass 60 Da to 20 Da below precursor mass, lowest precursor 707.46 Da, peak density filter 50 per 200 Da, maximum number peaks 65, fixed modification carbamidomethyl (C), variable modification oxidation (M), fragment ion tolerance 0.3, precursor tolerance 0.5. Proteins identified by the MASCOT algorithm were filtered based on proteins identified with MS/MS spectra, protein score CI >95%, protein score >69, proteins with isoelectric points (PI) and molecular weights (MW) that match the gel spot. Click here for file

Additional file 2 **Validation of identified *Torpedo *proteins in spectral data using Public Access Databases**. *Torpedo *protein sequences listed in GenBank were collected in a single database to directly search *Torpedo *electric organ fractions with a *Torpedo*-specific protein database. Results include *Torpedo *proteins also identified by a search against UniProtKB, all species. UniProtKB accessions are used for consistency. The chart categorizes proteins positively identified, not identified that are expected to be found with a possible explanation, and proteins not identified that are not expected to be identified. Reviewed sequences are from the UniProtKB/Swiss-Prot database and unreviewed sequences from UniProtKB/TrEMBL. Click here for file

Additional file 3 ***Torpedo californica *electric organ proteome classified according to tissue expression or associated function**. All proteins identified by mass spectral mapping (listed in Additional file 1) were queried in UniProtKB for annotation of tissue expression and or function then categorized as electric organ (EO) specific, neuromuscular junction (NMJ) specific, muscle specific or highly expressed in muscle, likely expressed in muscle based on function, expressed in neurons, or functions in metabolism and energy production. *Torpedo *cDNA sequences were queried by blastx (standard genetic code, Swiss-Prot, default algorithm parameters except for BLOSSUM80 scoring matrix) to obtain a protein identification with high sequence similarity. Click here for file

Additional file 4 **Neuromuscular junction (NMJ) proteins from the literature categorized by the degree of influence on synapse architecture**. The NMJ proteome was defined by searching the current literature and categorizing the influence of proteins on the synaptic structure and function. Category 1 represents mainly loss of function resulting in aberrant acetylcholine receptor (AChR) clustering and NMJ morphology and may result in an embryonic lethal. Category 2 represents interacting partners or dependencies with proteins in category 1. Category 3 represents proteins that are located at the NMJ but do not alter synapse morphology or function. Click here for file

Additional file 5 **Resolved *Torpedo californica *electric organ fractions**. *T. californica *electric organ fractions were resolved by one-dimensional electrophoresis on: **(a) **3% to 8% Tris-Acetate Novel NuPage^® ^MidiGel and **(b) **4% to 12% Bis-Tris Novel NuPage^® ^MiniGel. **(c) **The membrane-rich protein fraction from a lipid raft assay was resolved one dimensionally (4% to 12% Bis-Tris Novel NuPage^® ^MiniGel). Protein bands were excised (shown in black), subjected to in-gel trypsin digestion, and analyzed by nanospray electrospray ionization quadrupole linear ion-trap tandem mass spectrometry (ESI-LTQ MS/MS) analysis. Identification of proteins was performed using BioWorks 3.3.1 to crosscorrelate a combination of cross-species and *Torpedo *cDNA MS/MS spectral matching. **(d) ***T. californica *electric organ cytosolic fraction was resolved two dimensionally (IPG pH 3-10 and 8% to 16% CriterionTris-HCl Linear Gradient SDS gel). Protein spots were excised, subjected to in-gel trypsin digestion, and analyzed by matrix-assisted laser desorption/ionization - time of flight/time of flight mass spectrometry (MALDI-TOF/TOF MS). Identification of proteins was performed by GPS Explorer software to search spectra against UniProtKB/Swiss-Prot via MASCOT. Click here for file
