# Supplementary material for: Integrated genomics and proteomics of the Torpedo californica electric organ: concordance with the mammalian neuromuscular junction
Source: Skelet Muscle. 2011 May 4;1:20. doi: 10.1186/2044-5040-1-20 (PMC3156643; doi:10.1186/2044-5040-1-20)
Supplement: Additional file 4 — Neuromuscular junction (NMJ) proteins from the literature categorized by the degree of influence on synapse architecture. The NMJ proteome was defined by searching the current literature and categorizing the influence of proteins on the synaptic structure and function. Category 1 represents mainly loss of function resulting in aberrant acetylcholine receptor (AChR) clustering and NMJ morphology and may result in an embryonic lethal. Category 2 represents interacting partners or dependencies with proteins in category 1. Category 3 represents proteins that are located at the NMJ but do not alter synapse morphology or function. [file 2044-5040-1-20-S4.DOC]

| **Category** | **Protein** | **Reference** |
| --- | --- | --- |
| 1 | Lrp4 | Weatherbee SD, Anderson KV, Niswander LA**: LDL-receptor-related protein 4 is crucial for formation of the neuromuscular junction.** *Development* 2006, 133:4993-5000. |
| 1 | MuSK | Hesser BA, Henschel O, Witzemann V**: Synapse disassembly and formation of new synapses in postnatal muscle upon conditional inactivation of MuSK.** *Mol. Cell. Neurosci.* 2006, 31:470-80. |
| 1 | Rapsyn | Gautam M, Noakes PG, Mudd J, Nichol M, Chu GC, Sanes JR, Merlie JP: **Failure of postsynaptic specialization to develop at neuromuscular junctions of rapsyn-deficient mice.** *Nature* 1995, 377:232-236. |
| 1 | β-spectrin | Bloch RJ, Morrow JS: **An Unusual β-Spectrin Associated with Clustered Acetylcholine Receptors.** *J. Cell. Biochem.*1989, 108:481-493. |
| 1 | Dok7 | Okada K, Inoue A, Okada M, Murata Y, Kakuta S, Jigami T, Kubo S, Shiraishi H, Eguchi K, Motomura M, Akiyama T, Iwakura Y, Higuchi O, Yamanashi Y**: The Muscle Protein Dok-7 Is Essential for Neuromuscular Synaptogenesis.** *Science* 2006, 312:1802-1805. |
| 1 | Src and Fyn Kinase | Sadasivam G, Willmann R, Lin S, Erb-Vögtli S, Kong XC, Rüegg MA, Fuhrer C**: Src-Family Kinases Stabilize the Neuromuscular Synapse In Vivo via Protein Interactions, Phosphorylation, and Cytoskeletal Linkage of Acetylcholine Receptors.** *J. Neurosci.* 2005, 25:10479-10493. |
| 1 | Dvl | Luo ZG, Wang Q, Zhou JZ, Wang J, Luo Z, Liu M, He X, Wynshaw-Boris A, Xiong WC, Lu B, Me L: **Regulation of AChR Clustering by Dishevelled Interacting with MuSK and PAK1.** *Neuron* 2002, 35:489–505. |
| 1 | Rac | Weston C, Gordon C, Teressa G, Hod E, Ren X, Prives J**: Cooperative Regulation by Rac and Rho of Agrin-induced Acetylcholine Receptor Clustering in Muscle Cells.** *J. Biol. Chem.* 2003, 278:6450–6455. |
| 1 | ErbB2 | Lin W, Sanchez HB, Deerinck T, Morris JK, Ellisman M, Lee K**: Aberrant development of motor axons and neuromuscular synapses in erbB2-deficient mice**. *Proc. Natl. Acad. Sci. U.S.A.* 2000, 97:1299-1304. |
| 1 | LAMB2 | Noakes PG, Gautam M, Mudd J, Sanes JR, Merlie JP**: Aberrant differentiation of neuromuscular junctions in mice lacking s-laminin/laminin beta 2.** *Nature* 1995, 374:258-262. |
| 1 | PKC | Lanuza MA, Li MX, Jia M, Kim S, Davenport R, Dunlap V, Nelson PG**: Protein Kinase C-Mediated Changes in Synaptic Efficacy at the Neuromuscular Junction In Vitro: The Role of Postsynaptic Acetylcholine Receptors.** *J. Neurosci. Res.* 2000, 61:616–625. |
| 1 | pofut1 | Kim ML, Chandrasekharan K, Glass M, Shi S, Stahl MC, Kaspar B, Stanley P, Martin PT**: O-fucosylation of muscle agrin determines its ability to cluster acetylcholine receptors.** *Mol. Cell. Neurosci.* 2008, 39:452–464. |
| 2 | agrin | Gautam M, Noakes PG, Moscoso L, Rupp F, Scheller RH, Merlie JP, Sanes JR**: Defective neuromuscular synaptogenesis in agrin-deficient mutant mice.** *Cell* 1996, 85:525-536. |
| 2 | laminin subunits α4, α5 | Nishimune H, Valdez G, Jarad G, Moulson CL, Müller U, Miner JH, Sanes JR**: Laminins promote postsynaptic maturation by an autocrine mechanism at the neuromuscular junction.** *J. Cell Biol.* 2008, 182:1201–1215. |
| 2 | HSP90β and HSP70 | Luo S, Zhang B, Dong XP, Tao Y, Ting A, Zhou Z, Meixiong J, Luo J, Chiu FC, Xiong WC, Mei L: **HSP90 beta regulates rapsyn turnover and subsequent AChR cluster formation and maintenance.** *Neuron* 2008, 60:97–110. |
| 2 | α-syntrophin | Adams ME, Kramarcy N, Krall SP, Rossi SG, Rotundo RL, Sealock R, Froehner SC**: Absence of a-Syntrophin Leads to Structurally Aberrant Neuromuscular Synapses Deficient in Utrophin.** The *J. Cell Biol.* 2000, 150:1385–1397. |
| 2 | PAK1 | Luo ZG, Wang Q, Zhou JZ, Wang J, Luo Z, Liu M, He X, Wynshaw-Boris A, Xiong WC, Lu B, Me L: **Regulation of AChR Clustering by Dishevelled Interacting with MuSK and PAK1.** *Neuron* 2002, 35:489–505. |
| 2 | Rho | Weston C, Gordon C, Teressa G, Hod E, Ren X, Prives J**: Cooperative Regulation by Rac and Rho of Agrin-induced Acetylcholine Receptor Clustering in Muscle Cells.** *J. Biol. Chem.* 2003, 278:6450–6455. |
| 2 | cdk5 | Fu AK, Ip FC, Fu WY, Cheung J, Wang JH, Yung WH, Ip NY**: Aberrant motor axon projection, acetylcholine receptor clustering, and neurotransmission in cyclin-dependent kinase 5 null mice.** *Proc. Natl. Acad. Sci. U.S.A.* 2005, 102:15224–15229. |
| 2 | 14-3-3γ | Strochlic L, Cartaud A, Mejat A, Grailhe R, Schaeffer L, Changeux JP, Cartaud J**: 14-3-3γ associates with muscle specific kinase and regulates synaptic gene transcription at vertebrate neuromuscular synapse.** *Proc. Natl. Acad. Sci. U.S.A.* 2004, 101:18189–18194. |
| 2 | dynamin and NSF | Zhu D, Yang Z, Luo Z, Luo S, Xiong WC, Mei L**: Muscle-Specific Receptor Tyrosine Kinase Endocytosis in Acetylcholin Receptor Clustering in Response to Agrin.** *J. Neurosci.* 2008, 28:1688-1696. |
| 2 | ephexin1 | Shi L, Butt B,Ip FCF, Dai Y, Jiang L, Yung WH, Greenberg ME, Fu A, Ip NY**: Ephexin1 Is Required for Structural Maturation and Neurotransmission at the Neuromuscular Junction.** *Neuron* 2010, 65:204-216. |
| 2 | α-actinin | Dobbins GC, Luo S, Yang Z, Xiong WC, Mei L: **Alpha-Actinin interacts with rapsyn in agrin-stimulated AChR clustering.** *Mol. Brain* 2008, 1:1-18. |
| 2 | utrophin, neurogulin, ets transcription factor GABP | Hughes BW, Kusner LL, Kaminsky HJ**: Molecular Architecture of the Neuromuscular Junction.** *Muscle Nerve* 2006, 33:445-461. |
| 2 | Ras, Raf, MEK, MKK4, JNK, c-Jun | Mejat A, Ravel-Chapuis A, Vandromme M, Schaeffer L**: Synaptic-specific Gene Expression at the Neuromuscular Junction.** *Ann. N.Y. Acad. Sci.* 2003, 998:53-65. |
| 3 | ankyrin, desmin, α-dystrobrevin, vinculin, talin, paxilin, filamin | Froehner SC: **The submembrane machinery for nicotinic acetylcholine receptor clustering.** *J. Cell. Biochem.* 199, 114:1 1-7. |
