# Supplementary figures and images for: Integrated genomics and proteomics of the Torpedo californica electric organ: concordance with the mammalian neuromuscular junction
Source: Skelet Muscle. 2011 May 4;1:20. doi: 10.1186/2044-5040-1-20 (PMC3156643; doi:10.1186/2044-5040-1-20)

A

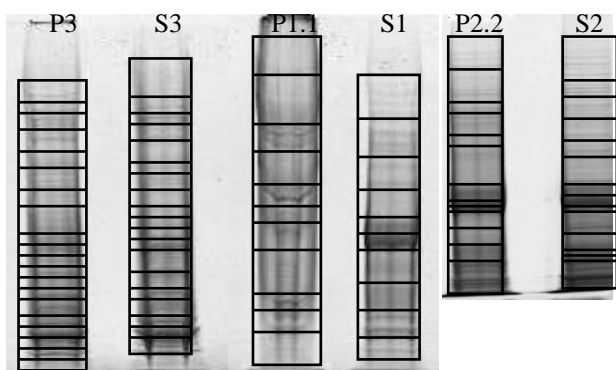

B

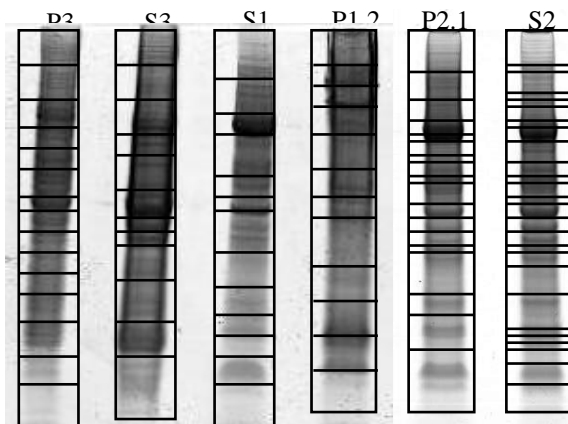

C

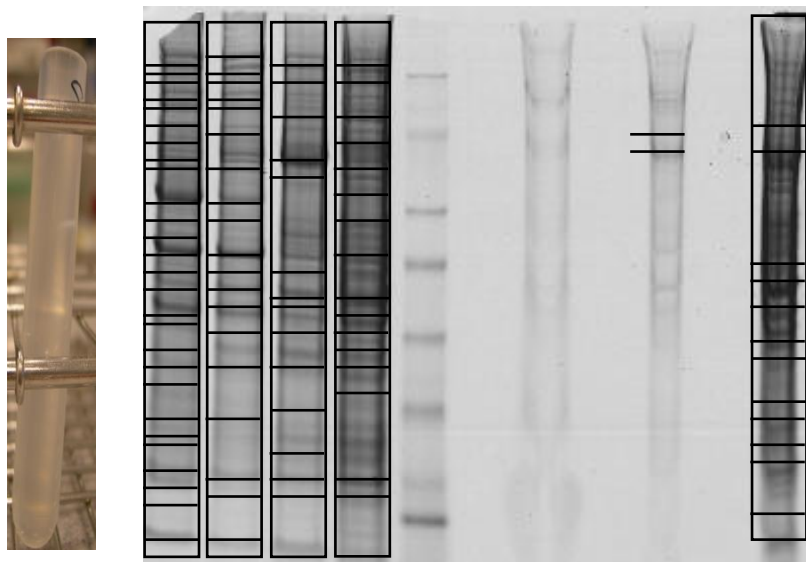

D

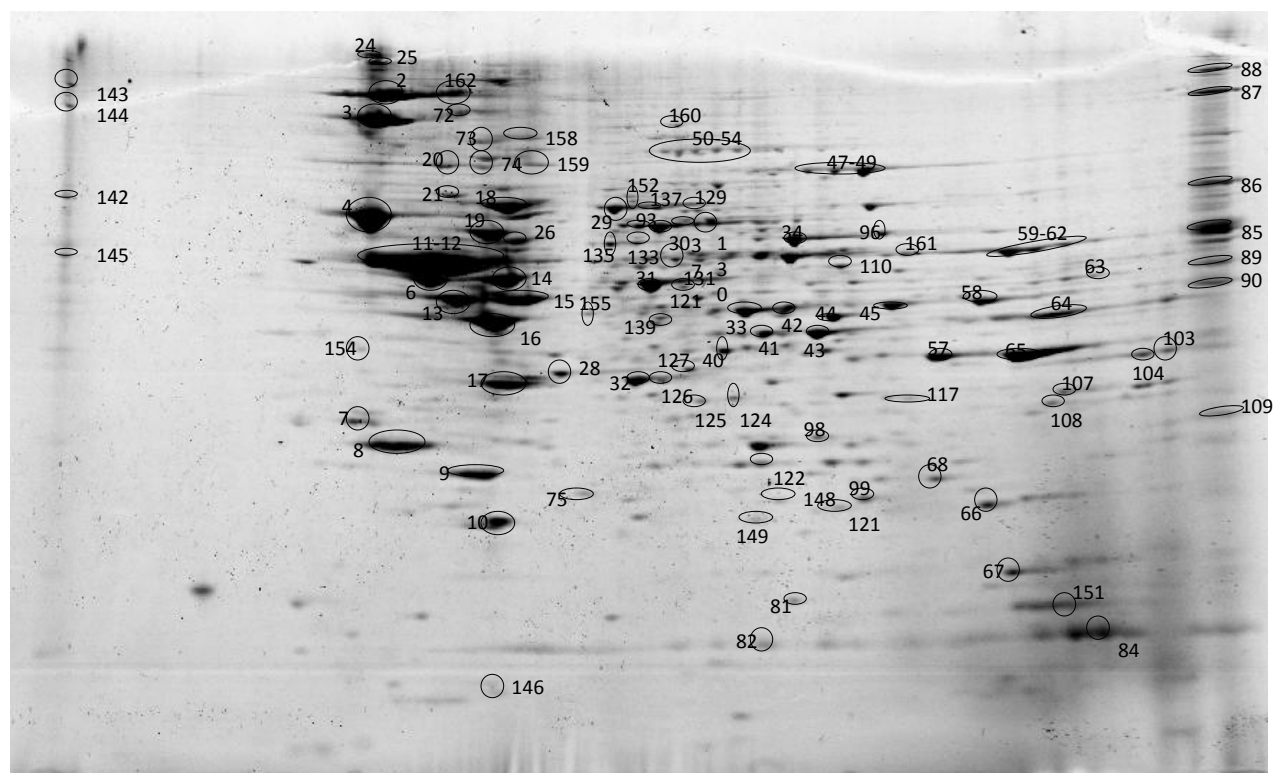

Supplement: Additional file 5 — Resolved Torpedo californica electric organ fractions. T. californica electric organ fractions were resolved by one-dimensional electrophoresis on: (a) 3% to 8% Tris-Acetate Novel NuPage® MidiGel and (b) 4% to 12% Bis-Tris Novel NuPage® MiniGel. (c) The membrane-rich protein fraction from a lipid raft assay was resolved one dimensionally (4% to 12% Bis-Tris Novel NuPage® MiniGel). Protein bands were excised (shown in black), subjected to in-gel trypsin digestion, and analyzed by nanospray electrospray ionization quadrupole linear ion-trap tandem mass spectrometry (ESI-LTQ MS/MS) analysis. Identification of proteins was performed using BioWorks 3.3.1 to crosscorrelate a combination of cross-species and Torpedo cDNA MS/MS spectral matching. (d) T. californica electric organ cytosolic fraction was resolved two dimensionally (IPG pH 3-10 and 8% to 16% CriterionTris-HCl Linear Gradient SDS gel). Protein spots were excised, subjected to in-gel trypsin digestion, and analyzed by matrix-assisted laser desorption/ionization - time of flight/time of flight mass spectrometry (MALDI-TOF/TOF MS). Identification of proteins was performed by GPS Explorer software to search spectra against UniProtKB/Swiss-Prot via MASCOT. [file 2044-5040-1-20-S5.PDF]
